# The ground beetle *Pseudoophonus rufipes* gut microbiome is influenced by the farm management system

**DOI:** 10.1038/s41598-022-25408-7

**Published:** 2022-12-31

**Authors:** Serena Magagnoli, Daniele Alberoni, Loredana Baffoni, Antonio Martini, Francesca Marini, Diana Di Gioia, Martina Mazzon, Claudio Marzadori, Gabriele Campanelli, Giovanni Burgio

**Affiliations:** 1 Dipartimento di Scienze e Tecnologie Agro-Alimentari (DISTAL), Università di Bologna, Viale Fanin 50, 40127 Bologna, Italy; 2 Consiglio per la ricerca in agricoltura e l’analisi dell’economia – Centro di ricerca Orticoltura e Florovivaismo (CREA -OF) - Sede di Monsampolo del Tronto, via Salaria 1, 63077 Monsampolo del Tronto, Italy

**Keywords:** Bioinformatics, Biological models, Microbiology techniques, Biological techniques, DNA sequencing, Next-generation sequencing, Agroecology, Biodiversity, Ecosystem ecology, Ecosystem services, Microbial ecology, Theoretical ecology

## Abstract

Intensive conventional farm management, characterized by high agrochemicals input, could alter the composition of microbial communities with potential negative effects on both functional traits and the ecosystem services provided. In this study, we investigated the gut microbial composition of a high ecological relevance carabid *Pseudoophonus rufipes*, sampled in two fields subjected to conventional and organic management practices. Carabids’ gut microbiota was analyzed via qPCR and NGS. Profound differences between the microbial composition of organic and conventional samples were detected: the abundance of Tenericutes and Proteobacteria was significant higher in organic and conventional samples, respectively. Spiroplasmataceae and Bifidobacteriaceae families were significantly more abundant in samples from organic management, while Enterococcaceae, Morganellaceae and Yersiniaceae were more abundant in samples from conventional management. The diverse gut microbial composition of insects between the two management systems is related to the pressure of environmental stressors and it may representing an important bioindication of ecological functions and services provided by a carabid species.

## Introduction

Gut microorganisms play a relevant role in insect life, with functional support in the host’s metabolism of nutrients, immune stimulation and modulation, protection from pathogens, xenobiotic detoxification^1,2^. The bacterial diversity in insect digestive tracts is generally lower than that recorded in mammals and is affected by host species and the life stage, diet and environmental drivers^3–5^. The gut microbiota of the herbivore insects has been studied for important aspects linked to the biosynthesis of essential nutrients and the plant biomass digestion^1,6^. These studies, in the scenario of ‘omics’ age, were later followed by others on beneficial insect’s species involved in important ecosystem services like honey bees (*Apis mellifera* L.) and ground beetles, that were proposed as a model for the research on the gut microbiota^7,8^. The impact of xenobiotics and antibiotics of anthropic origin and the different environmental parameters on the honey bees gut microbiota was deeply investigated^4,9–12^, and their influence on the gut microbiome shaping and composition was outlined. In the same way, also some studies on ground beetles gut microbiota diversity association to diet and land use were proposed^13–15^.

Microbial diversity of insect gastrointestinal tracts can be linked to the biodiversity of the habitats where insects live, highlighting important implications as to how reductions in habitat biodiversity may affect the ecological functions and services that target species can perform^16^. In addition, the exposure to xenobiotics in agriculture can strongly influence the insect gut microbiota, as recent works have shown on different animal taxa^17^. For example, exposure of bees to pesticides^18^ and glyphosate^19^ can perturb and alter their beneficial gut microbiota. Other studies demonstrated an influence of landscape exposure on honey bee microbial community, highlighting the potential effect of different environmental parameters, such as land cover (pollen and nectar sources)^4^ and neonicotinoid pesticides^20^ on key honey bee gut bacteria. Recently, a study revealed that pendimetalin-based herbicide can impact microbial communities associated with a ground beetle generalist predator^21^.

Biodiversity conservation is an important goal of sustainability, and organic farming systems are considered potential solutions to the loss of diversity in agricultural landscape and the associated ecosystem services^22–24^. The concept of multifunctionality is based on the ability of ecosystems to simultaneously provide multiple services. This holistic approach has become increasingly common in recent years thanks to the results obtained within collaborative projects between different disciplines and teams. The European Union has made “multifunctionality” its primary objective for agriculture, in order to protect the environment and rural heritage^25,26^. Farm management, ranging from the more intensive conventional to the sustainable organic, could affect the gut microbiome of insect species involved in important ecosystem services. As far as we know, little is known about the influence of farm management strategies on the gut microbiome of terrestrial beneficial arthropods. Intensive conventional management, providing high xenobiotic input (i.e. pesticides, herbicide and fungicides) could cause alteration in the composition of the insect gut microbial communities, with consequent potential changes in functional traits and on the ecosystem services provided. In this context, gut microbial community could be a sensitive parameter, providing a bioindication of ecological sustainability of a farm.

In the light of above considerations, this field research was carried out to evaluate whether farming management strategies (organic vs conventional) can have an impact on the Carabidae gut microbiome in an experimental farm of the Mediterranean area. Within the farm area an organic (aprox. 0.65 ha) and a conventionally managed field (aprox. 0.42 ha) 300 m distance each other were considered. Both the fields were characterized by a Typic Calcixerepts fine-loamy, mixed thermic soil^27^. *Pseudoophonus rufipes* (De Geer) (^28^ Coleoptera; Carabidae) were selected for their importance in providing standard bioindication^29^ and important ecosystem services like pest predation and seed consumption in agriculture^30,31^.

The study site, a long-term experimental farm, was selected for the availability of agronomic^32,33^ and soil diversity data, recorded in previous studies^34,35^. Specifically, this work aims to determine if agricultural management practices affect *P. rufipes* gut microbiome, leading to the possible use of this insect microbiome as an environmental degradation level indicator. *Pseudoophonus rufipes* microbiome may be used as model of ecological sustainability of farm management.

## Results

### Collected carabids

A total of 50 alive Carabid specimens were collected, including three species: *P. rufipes*^28^ (58%), *Poecilus cupreus* (L.) (36%) and *Pterostichus melas italicus* (Dejean) (6%). Abundances of specimens collected in conventional were significantly lower than those in organic; this trend agrees with previous studies in the same long-term experimental farm, which demonstrated a richer and abundant soil fauna (including Carabids) in the organic rotation in comparison with conventional one^34^. *Pseudoophonus rufipes* (N = 29) was selected because it was the dominant specie, in accordance with the previous study employed in the same experimental farm^34^; moreover, this species displayed a more even ratio between the two management regimes.

### NGS results

**Figure 1:**
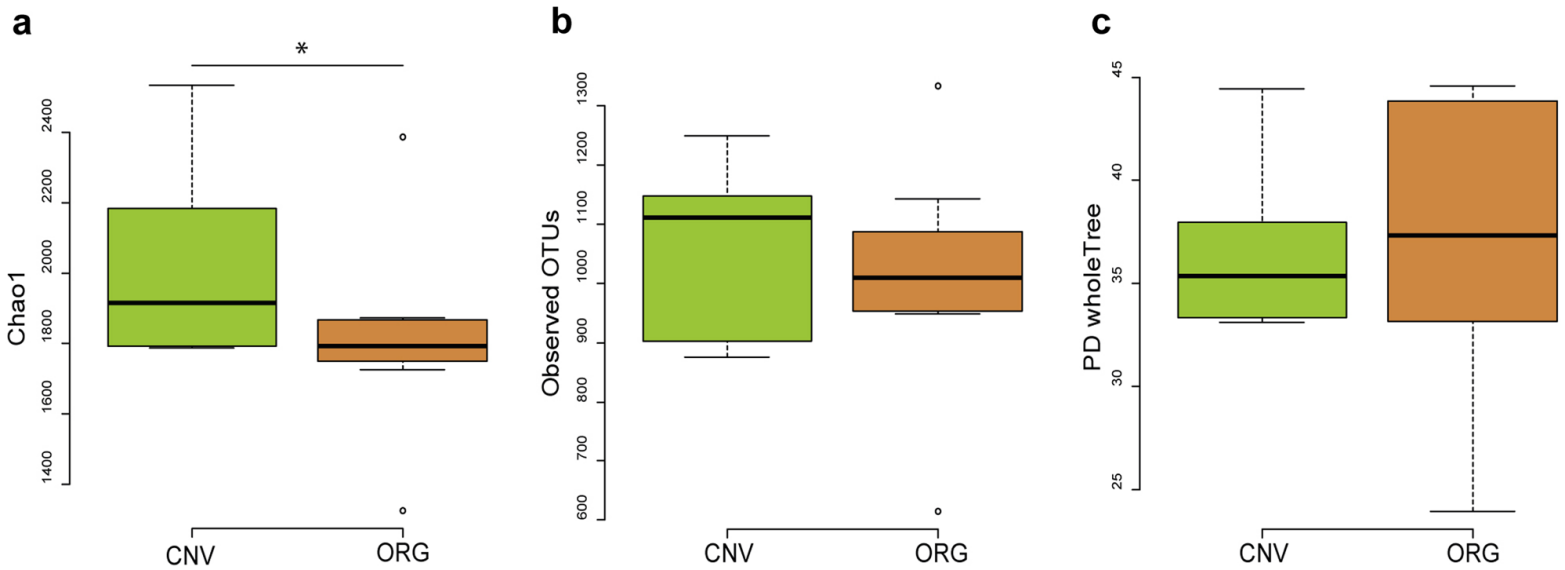
α-Diversity indexes. (**a**) Chao1; (**b**) Observed OTUs; (**c**) and PD Whole Tree. Significant pairwise comparisons *p < 0.05. [CNV] Conventional managment; [ORG] organic managements

About 2.5 million raw reads were obtained from sequencing, 1.9 million of which passed the quality control and the chimera check analysis with an average of 53k joint reads per sample (ranging from 33,717 to 81,006). The α-diversity index Chao1 showed a significant reduction of diversity (*p* = 0.051; Fig. 1a) in ORG when compared to CNV experimental condition. Observed OTU and PD whole tree on the same dataset did not show a significant difference in α-diversity (Fig. 1b and 1c, respectively). The β-diversity index weighted UniFrac did not show significant differences (Table S1). However, unweighted UniFrac (Table S1), displayed statistically significant differences between CNV and ORG treatments (*P* > 0.05).

### Analyses of phyla

**Figure 2:**
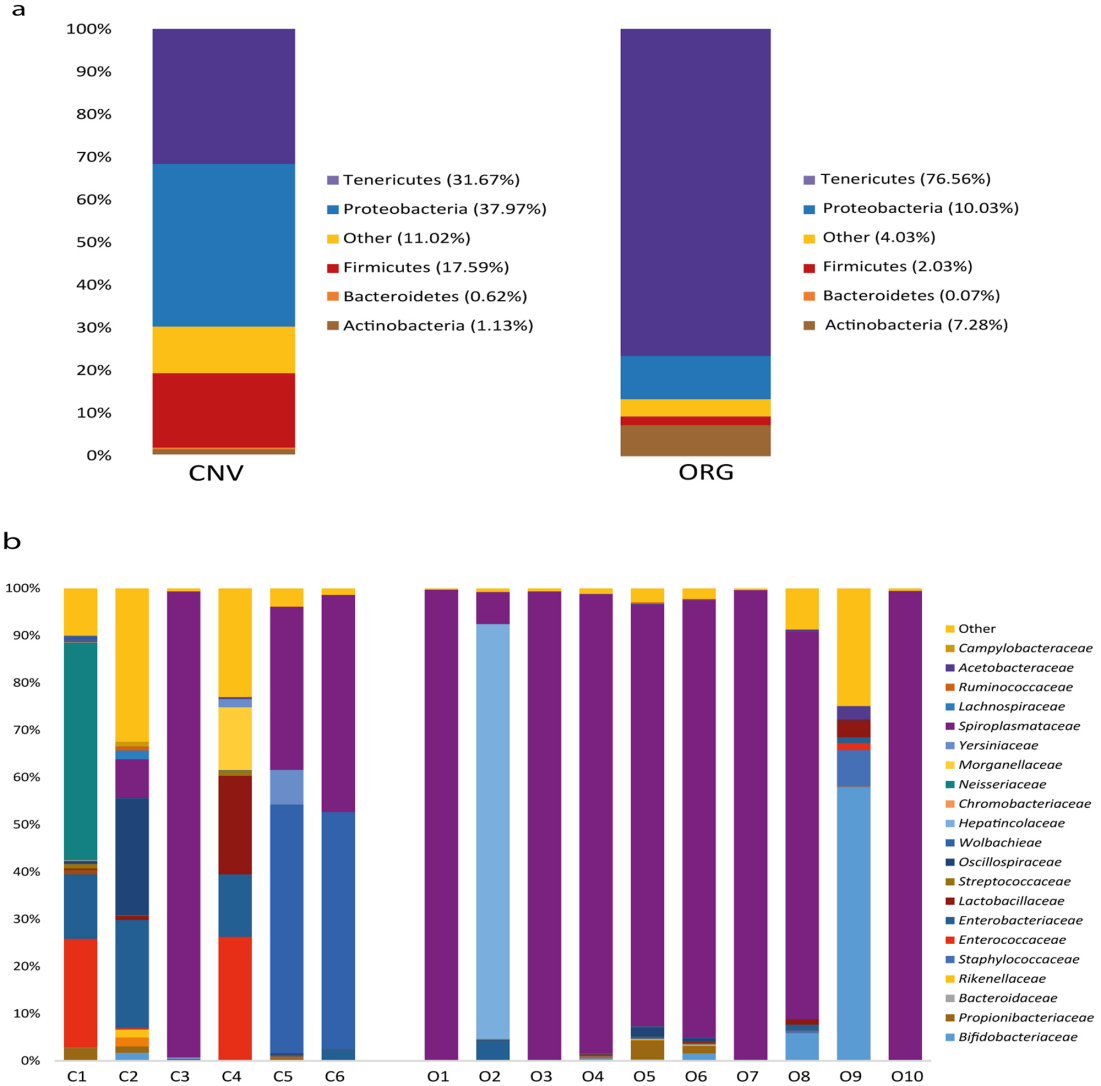
Taxonomy at Phylum and Family level. Barcharts showing the relative abundance of *P. rufipes* gut microbial communities at phylum level (**a**) and family level (**b**) in the conventional [CNV] and organic [ORG] managements.

A total of 15 phyla were recorded in *P. rufipes* samples. The most representative phyla in both management practices were: Tenericutes (average 31.67% in CNV and 76.56% in ORG), Protobacteria (average 37.97% in CNV and 10.03% in ORG), Firmicutes (average 17.59% in CNV and 2.03% in ORG), Actinobacteria (average 1.13% in CNV and 7.28% in ORG) and Bacteroidetes (average 0.62% in CNV and 0.07% in ORG) (Fig. 2), while other phyla accounted for very low abundance (< 0.5%).

**Figure 3:**
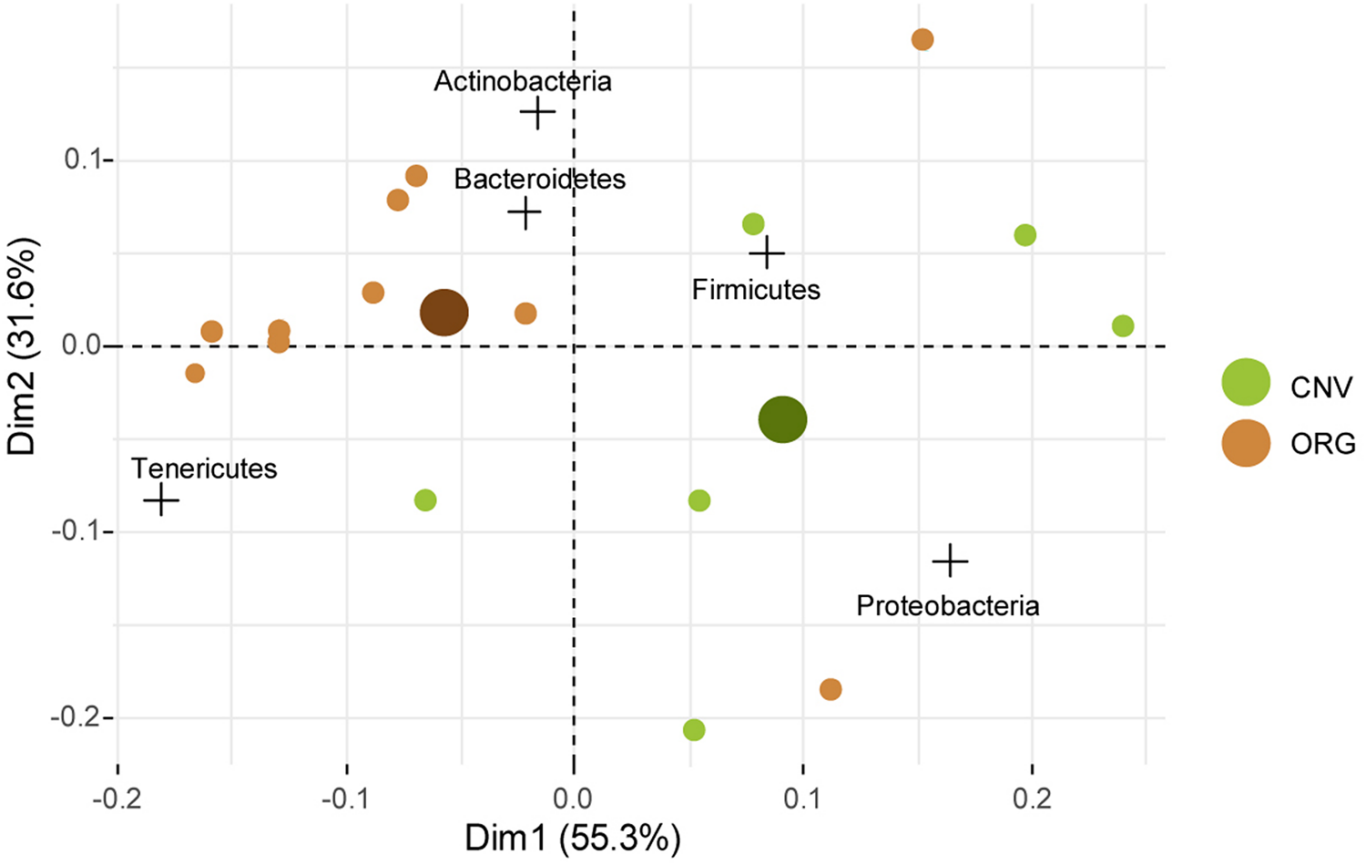
Correspondence analysis of phyla. [ORG] Organic management and, [CNV] Conventional management samples. CNV and ORG indicate centroid of conventional and organic, respectively.

**Figure 4:**
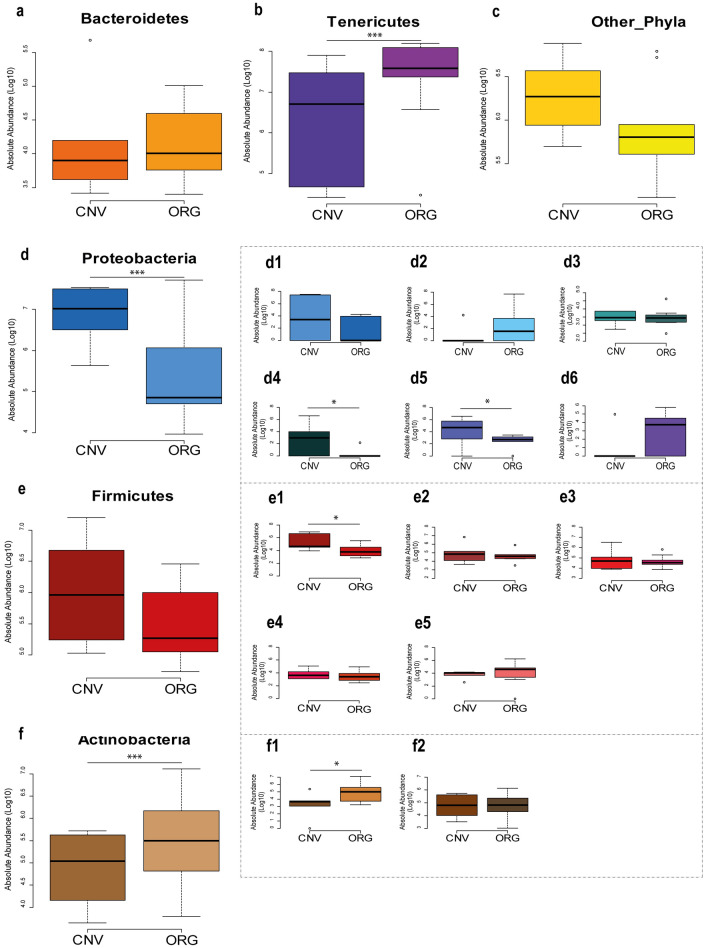
Relative abundance normalized with qPCR absolute quantification on total bacteria. Box plots report the major microbial phyla and families expressed for their relative abundance (qPCR normalized NGS relative abundance), and in relation to experimental conditions (conventional [CNV] and organic [ORG]; significant pairwise comparisons *p < 0.05; ***p < 0.01). Boxplots report minimum and maximum values, lower and upper quartile and median. Microbial Phyla described: (**a**) Bacteroidetes; (**b**) Tenericutes; (**c**) Other unclassified taxa; (**d**) Proteobacteria; (**e**) Firmicutes; (**f**) Actinobacteria. Microbial Families described: (d1) Wolbachieae; (d2) Hepatincolaceae; (d3) Neisseriaceae; (d4) Morganellaceae; (d5) Yersiniaceae; (d6) Acetobacteraceae; (e1) Enterococcaceae; (e2) Lactobacillaceae; (e3) Oscillospiraceae; (e4) Ruminococcaceae; (e5) Staphylococcaceae; (f1) Bifidobacteriaceae; (f2) Propionibacteriaceae. At Family level Spiroplasmataceae and Bacteroidaceae showed the same proportions of Tenericutes and Bacteroidetes respectively, therefore these families are not displayed in the figure. Other_Phyla is the sum of 9 minor phyla present in the *P. rufipes* gut.

PERMANOVA performed on absolute concentrations of phyla showed a statistical difference between ORG and CNV (*P* < 0.05). Correspondence analysis (CA) obtained on phyla showed a general differentiation of ORG samples from those of CNV ones; also, centroids of the two farm management practices were clearly separated. CA on phyla showed a total of explained variance by first two axes of 87% (first axis = 55%; second axis = 32%). Besides this general trend, some samples (O2 and C3) were grouped far from their own groups. Proteobacteria and Firmicutes were more correlated with CNV, while Tenericutes and Actinobacteria were closest to ORG (Fig. 3). Median test confirmed that Proteobacteria were significantly more represented in CNV, while Tenericutes were more abundant in ORG (*P* < 0.05) (Fig. 4).

### Analyses of families

At family level, about 240 families were detected in *P. rufipes* samples. Twenty-three main families plus an unclassified group belonging to the Mollicutes class were recorded in both management practices, but only 11 families showed an absolute abundance higher of 1% in at least one of the two experimental conditions. Of these families, only those with a relative abundance > 4% were statistically analysed. PERMANOVA detected significant differences of family composition between ORG and CNV (*P* < 0.01). Spiroplasmataceae was the dominant family, showing a relative abundance of 31.27% in CNV and 76.55% in ORG. Bifidobacteriaceae relative abundance was 0.30% in CNV and 6.65% in ORG, whereas Hepatincolaceae accounted for 0.01% in CNV and 8.79% in ORG. However, Bifidobacteriaceae were detected in 4 samples with an absolute abundance > 1%, one of which (sample 39Q) showed a relative abundance of 57.99%. Hepatincolaceae family was present with a relative abundance > 4% in just one sample (87,84%). The families that were more abundant in CNV were: Enterococcaceae (8.37% in CNV and 0.17% in ORG), Enterobacteriaceae (average 8.84% in CNV and 0.71% in ORG), Lactobacillaceae (average 3.69% in CNV and 0.53% in ORG), Oscillospiraceae (average 4.28% in CNV and 0.32% in ORG), Wolbachieae (average 17.15% in CNV and 0.01% in ORG), Neisseriaceae (average 7.92% in CNV and 0.02% in ORG), Morganellaceae (average 2.24% in CNV and 0.00% in ORG) and Yersiniaceae (average 7.92% in CNV and 0.02% in ORG) (Fig. 4).

**Figure 5:**
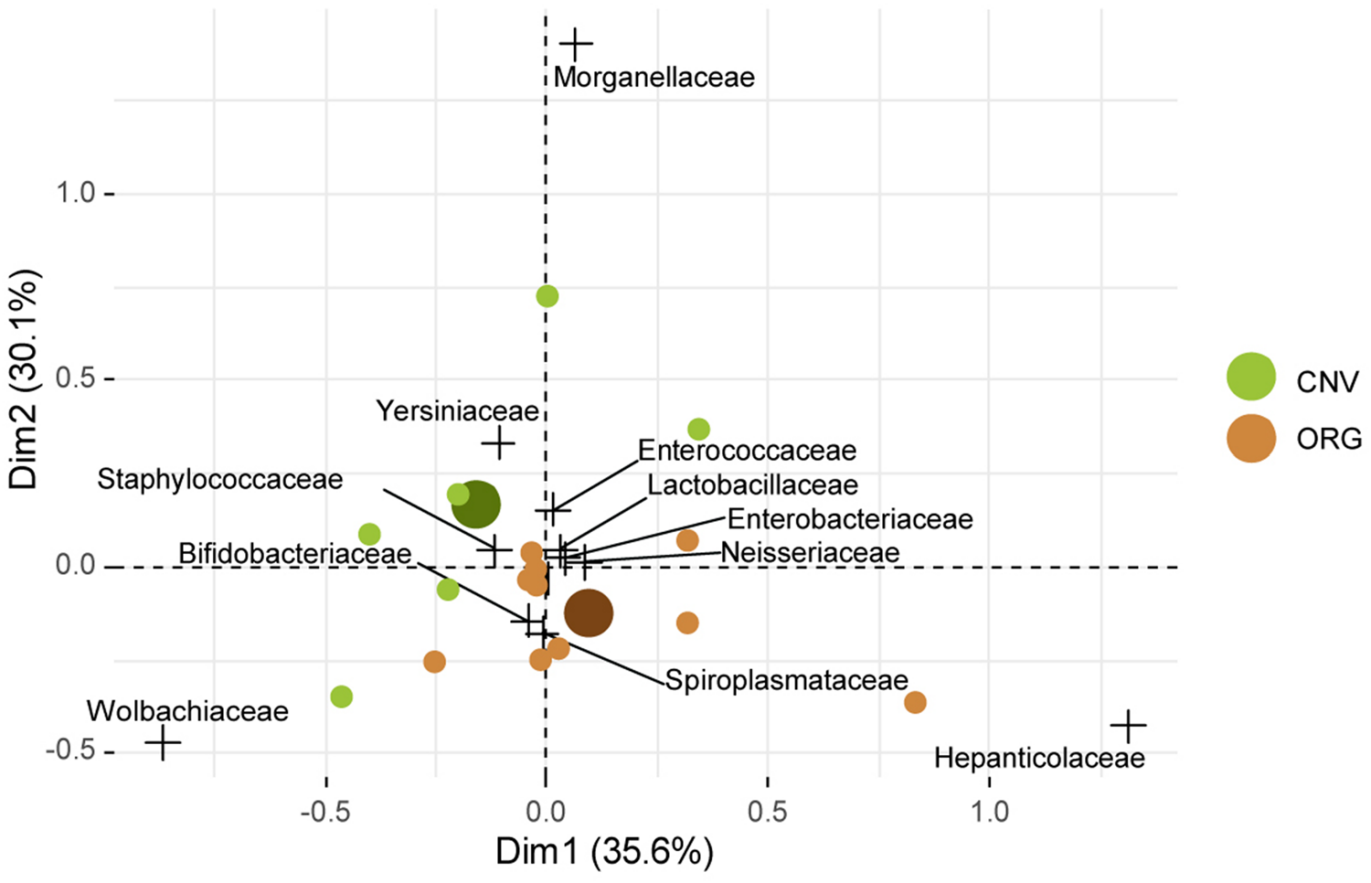
Correspondence analysis of families. [ORG] Organic management and [CNV] Conventional management samples. CNV and ORG indicate centroid of conventional and organic, respectively.

Correspondence analysis (CA) on absolute concentration of families confirmed that ORG and CNV samples clustered into different groups. Morganellaceae, Enterococcaceae, Yersiniaceae correlated with CNV, while Bifidobacteriaceae, Spiroplasmataceae, Hepatincolaceae were close to ORG; it should be noted that Hepatincolaceae correlated with ORG just by one sample (O2) (Fig. 5). A number of families showed an uncertain ordination, explained by variability of samples or a differential response of genera belonging to same families. Median test revealed a higher concentration of Enterococcaceae, Morganellaceae and Yersiniaceae in CNV than ORG (*P* < 0.05), while taxa like Bifidobacteriaceae and Spiroplasmataceae were more abundant in ORG than CONV (*P* < 0.05). The Indicator Value Analysis confirmed that Morganellaceae and Spiroplasmataceae taxa are significantly associated with CNV with ORG respectively (*p* < 0.05), while all the other families are not associated with a specific treatment.

### Analyses of genera

**Figure 6:**
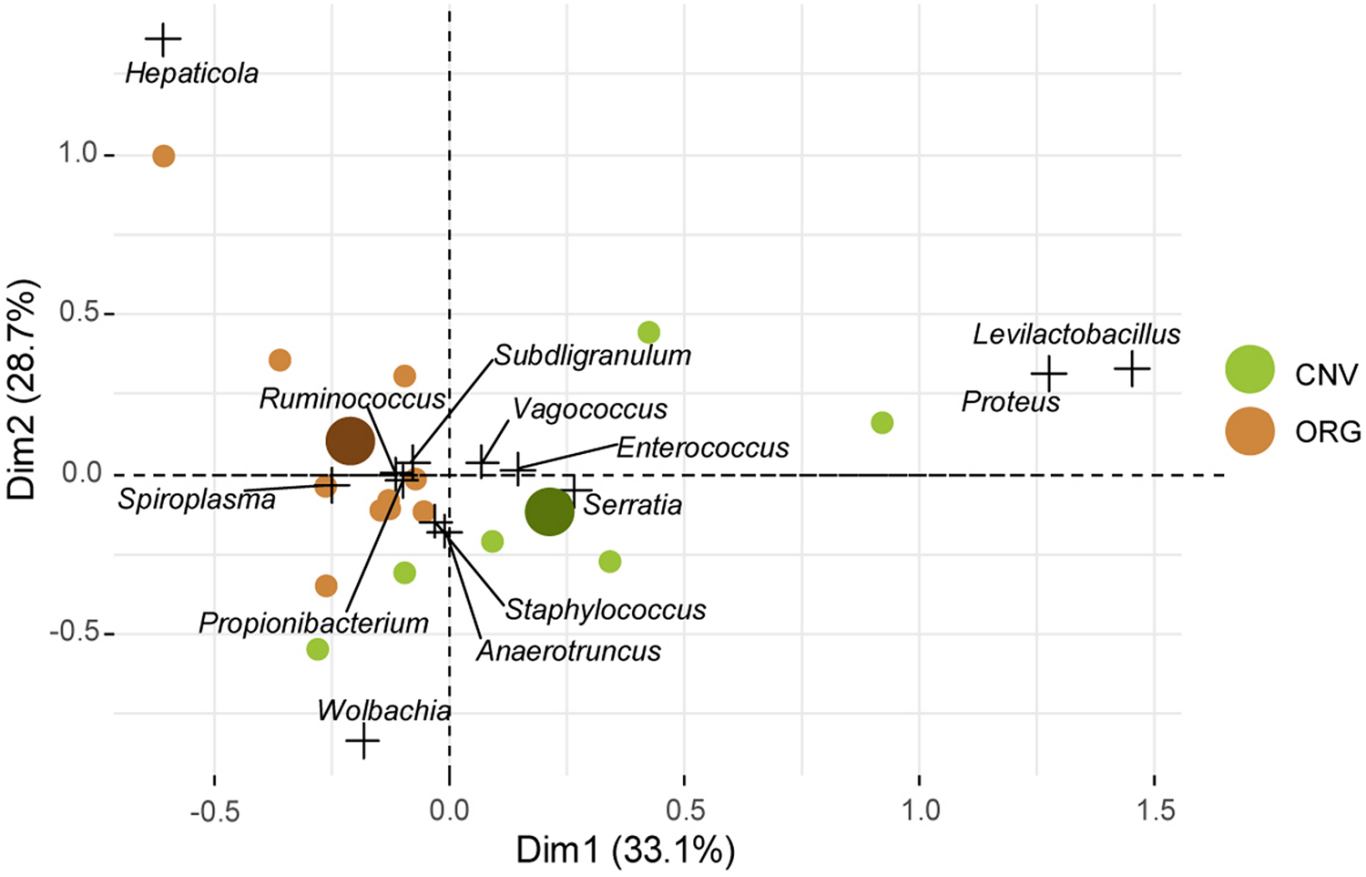
Correspondence analysis of genera.  [ORG] Organic management and [CNV] Conventional management samples. CNV and ORG indicate centroid of conventional and organic, respectively.

PERMANOVA performed on data related to genera having relative abundance higher than 4% showed statistical differences between ORG and CNV (*P* < 0.05; Fig. 6). At genus level, *Spiroplasma* was the dominant taxon of Spiroplasmataceae, with the exception of one CNV sample that showed 2.45% of an unclassified Mollicutes order. *Bifidobacterium* (0.30% in CNV and 6.15% in ORG) and *Gardnerella* (0.00% in CNV and average 0.36% in ORG) were the most representative genera of the Bifidobacteriaceae family.

Hepatincolaceae was present with the *Hepanticola* genus, while Enterococcaceae were mostly represented by *Enterococcus* (average 2.24% in CNV and 0.02% in ORG, *p* < 0.05) and *Vagococcus* (average 6.13% in CNV and 0.16% in ORG). Wolbachieae, Morganellaceae and Yersiniaceae were represented at genus level only by *Wolbachia*, *Proteus* and *Serratia*, respectively, which showed the same proportions described at family level.

Genera like *Citrobacter*, *Enterobacter* and *Raoultella* (Enterobacteriaceae) were represented with a very low relative abundance in both experimental conditions. *Levilactobacillus* was the only Lactobacillaceae genus with a relevant relative abundance (2.88% in CNV and 0.00% in ORG). Other detected Lactobacillaceae were *Lactiplantibacillus*, *Lactobacillus*, *Lactococcus*, and *Ligilactobacillus* although not relevant in relative proportion. Within Oscillospiraceae, *Ruminococcus* and *Subdoligranum* accounted for an average of 1.52% and 0.89% in CNV and 0.13% and 0.17% in ORG, respectively. Most Neisseriaceae were unclassified at genus level.

Also, CA performed on genera, showed that ORG and CNV samples were respectively ordinated in different groups. *Spiroplasma* and *Bifidobacterium* genera were clearly associated with ORG, while the strong association of Hepatincola with ORG was actually determined by a single sample (O2). *Levilactobacillus, Serratia, Proteus* and *Enterococcus* were correlated with CNV, while a number of genera showed an uncertain ordination. Absolute concentrations of *Spiroplasma* (Spiroplasmataceae), *Bifidobacterium* and *Gardnerella* (Bifidobacteriaceae) were higher in ORG than CNV (*P* < 0.05, median test), while *Proteus* (Morganellaceae) and *Vagococcus* (Enterococcaceae) were more abundant in CNV than ORG (*P* < 0.05); *Levictobacillus* displayed a higher absolute concentration in CNV (2.88%) than ORG (0.00%), with a statistic value close to the significance level (*P* = 0.07). The Indicator Value Analysis at genera level showed Proteous and Spiroplasma taxa as significantly associated with CNV with ORG respectively (*p* < 0.05).

## Discussion

In the present study, the gut microbiota of the Carabidae *P. rufipes* collected in fields characterized by two different agricultural practices (ORG and CNV) has been studied for the first time with a NGS based approach, targeting the V3-V4 region of the 16S rRNA gene. The two experimental fields involved in this study are a long-term test (since 2001) to assess the impact of agronomic practices on biodiversity and crops productivity. However, this work relay on a single collection site and the results obtained are not completely representative but a preliminary investigation.

The analysis of the biodiversity indexes showed few significant differences between CNV and ORG. Chao1 displayed a significant trend of increasing diversity in CNV; however, this index is strongly weighted on rare taxa. Moreover, only the β-diversity index unweighted UniFrac showed significant differences between CNV and ORG. Unweighted UniFrac considers the presence/absence of microbial taxa without balancing them differently according to their abundance, therefore, it is sensitive to differences in low-abundance taxa when compared to Weighted UniFrac. On the contrary, α-diversity indexes like observed OTUs, that test microbial richness in each sample, did not show any significant difference between CNV and ORG.

The gut microbial composition showed a certain inter-individual variability among samples collected in each farm management (Fig. 2). This variability is often found within insects and animals in general, and it is exacerbated when the sampling is done in field with respect to laboratory reared insects^36^. Also, a limited number of samples in ORG and CNV diverged from the core microbiota of the respective group to which they belonged; this could depend on the relative proximity of the two long-term experiment fields involved in the comparison that may have determined translocation of some *P. rufipes* specimens from a field to another. On the other hand, the contiguity of the two fields guaranteed an inter-site microbiome homogeneity that is difficult to find if experimental fields are located at some distance from each other.

The results revealed profound differences in the specimens collected in the two fields, being Proteobacteria and Tenericutes the most abundant phylum in CNV and ORG, respectively.

Proteobacteria is usually among the dominant phyla in insects^37^ including the other Carabidae species in which the gut microbiota has been studied^21,38,39^. Therefore, the differences we have found in the beetles collected at the organic management fields is in line with the observations that correlate the insect gut microbiota shaping to the environment^40^ and perhaps to pathogen spread on food sources^41^. A recent study carried out on the same long-term field experiment area^27^, aimed at evaluating soil quality considering different chemical and biochemical parameters, demonstrated a higher soil quality and microbial metabolic activity in the ORG fields with respect to the CNV ones. The outlined differences in soil quality support the large variation in the microbiota composition that we observed in the two farming conditions. The differences outlined in the carabids microbiome may also be influenced by the high number of plant species involved in crop rotation in ORG (n = 9) compared to CNV (n = 3) (Table 1). However, according to Silver et al.^38^, different diets (from carnivorous to herbivorous) may have minor effects on gut microbial community of ground beetles, at least in a short-term diet. This makes it unlikely that plant diversity could cause the microbiota to diverge so much in the two environments.

**Table 1:** Agronomic practices for the two experimental areas (conventional vs organic) as reported in Mazzon et al. (2021).

	Management	
	Conventional	Organic
Crop rotation	Fennel (*Foeniculum vulgare* M.); Cauliflower (*Brassica oleracea* L.); Tomato (*Solanum lycopersicum* L.)	Faba for fresh pod (*Vicia faba* L.) and after on the same soil (*Solanum lycopersicum* L.) in strip cropping with faba for dry grain (*Vicia faba* L.); Zucchini (*Cucurbita pepo* L.) in strip cropping with common wheat for dry grain (*Triticum aestivum* L.); Fennel (*Foeniculum vulgare* M.); Lattuce (*Lactuca sativa* L.); Cauliflower (*Brassica oleracea* L.); Bean (*Phaseolus vulgaris* L.)
Cover crops	None	Common wheat before zucchini; radish (*Raphanus sativus* L.) before lettuce
Tillage	Plowing (0.4 m); Harrowing (0.3–0.4 m)	No tillage
Irrigation	Drip system	Drip system
Weed controls	Bioplastic film and herbicides	Natural mulching (cover crop flattened)
Nutrients	Synthetich and mineral NPK fertilizers: Nitrofoska (12-12-12), 1000 kg/ha; NH_4_NO_3_ (26-0-0), 230 kg/ha	None
Crop at sampling time	*Solanum lycopersicum* L. *Cultivar* SAAB-CRA	*Solanum lycopersicum* L. *Cultivar* SAAB-CRA

Tenericutes are bacteria lacking the peptidoglycan cell wall and are therefore commensal of insects or obligate plant parasites^42^. Culture-independent high throughput sequencing techniques have allowed the detection of members of this phylum in several new habitats, showing a great adaptation capability inside the gut with the expression of metabolic pathways, such as acetate production, typical of this environment^42^. Tenericutes may have found in insects living and feeding in ORG fields suitable conditions to develop and replace the proteobacteria commensal population. Within the Tenericutes phylum, Spiroplasmataceae was the dominant family in both farming condition with a significant higher abundance in ORG samples than in CNV. This increase could simply depend on the lower content of other taxa in this farm management, leading to a rebalancing of microbial community or may be linked to a better state of the immune system of those carabids. This last assumption is based on the consideration that many *Spiroplasma* species have been shown to play an important role in host protection by increasing defenses against nematode parasites and wasp parasitoids^1^. In detail, it seems that *Spiroplasma* can directly interact with secreted molecules of the immune response involved in the important mechanisms of protection^43^. On the contrary, Enterococcaceae and Enterobacteriaceae were abundant in CNV, although their percentage was lower with respect to Spiroplasmataceae. Instead, Enterobacteriaceae was the most abundant taxon in *P. melas italicus* in the experiment of Giglio et al.^21^.

In the mentioned study on gut microbial community response to pendimetalin exposure in the ground beetle *P. melas italicus*, it was shown an increase in taxa like *Enterobacter* (Enterobacteriaceae), *Pseudomonas* (Pseudomonadaceae), *Pantoea* (Erwiniaceae, recently separated from *Enterobacter*) and *Paracoccus* (Rodobacteraceae) that may be involved in herbicide degradation. Moreover, authors revealed that herbicide exposure had effects on functional categories of gut microbiota related to important metabolic functions (i.e. metabolism of amino acids, lipids and carbohydrates). Since bacterial composition and diversity of Carabids have been seen largely resilient to controlled changes to host diet^38^, an increasing of some taxa in conventional (including Enterobacteriaceae) could be ascribed to stressors like pesticide use. Changes in the microbiota communities of the ground beetle *P. melas italicus* after exposition to an herbicide^21^ was likely the cause of variations of the antagonistic interaction among different bacteria with different metabolic requirements, including the increasing of facultative pathogens. Some physiological functions can be the product of interactions among microbial taxa, and the dominance of a species can be correlated negatively or positively with the abundance of other bacteria. Other cause-effect explanation, depending by physiological reasons, should be investigated. It would be noteworthy to investigate whether changes in the microbiome could cause changes in the ecosystem services provided by beneficial insects.

A different physiological condition could be affected by management regimes, leading, for example, to fluctuations in the intensity of biological control. Since the *P. rufipes* gut microbial composition differences between the two management systems may be related to the pressure of environmental stressors, the gut microbiome could represent and important bioindication of ecological functions (i.e. ORG against CNV) and/or characterize the fitness and the intrinsic quality of beneficial taxa that receive a different input of xenobiotics. It cannot be excluded that changes in gut microbiome may be due to the quality of the food and the kind of preys of an insect, that could be different between organic and conventional. Further studies should also explore the potential variability of the microbiome composition of species belonging to different agricultural management within different geographic areas; an interesting aspect should also be to confirm the stability of the dominant taxa found in this study and the correspondence between some bacteria taxa and a specific agronomic management. *Pseudoophonus rufipes* gut microbiome seems also scarcely affected by the soil microbiome, even if carabids are constantly in contact with soil, their natural habitat and nesting place^44^. The only two microbial families detected in the gut microbiome and shared with soil are Lactobaillaceae and Verrucomicrobiaceae, although the latter with low proportions.

Returning to the results of our study, the lower level of Enterobacteracae in ORG specimens may be correlated with a greater health status, which is also confirmed by the higher average percentage of Bifidobacteriaceae with respect to CNV collected specimens. Within Bifidobacteriaceae, *Bifidobacterium* was the dominant genus whereas *Gardnerella*, belonging to the same family, was found with low relative abundance. The beneficial role of *Bifidobacterium* members has been well documented with positive effects on immunomodulation and on the metabolism of oligosaccharides, ensuring an improved health status. Several genera of Bifidobacteriaceae have been found in honeybees^45^, bumblebees^46,47^ and carpenter bees^48^. However, this is the first report of *Bifidobacterium* detection, as far as we know, in a Carabid species; the functional role of this taxon in *P. rufipes* can be similar to those reported in other insect species and in depths studies, also aimed at isolation and species characterization, are envisaged. Taxa like Enterococcaceae, Morganellaceae and Yersiniaceae were prevalent in CNV: the genera that contributed to these differences were *Proteus* and *Vagococcus. Levilactobacillus* was dominant in CNV in comparison with ORG, although this difference was only close to the significance level. A *Levilactobacillus brevis* (basonym *Lactobacillus brevis*) strain isolated from kimchi, a fermented vegetable food traditionally prepared in Eastern countries, was found to be involved in chlorpyrifos degradation^49^. A similar role in degrading pesticide could be performed by the *Levictobacillus* hosted in *P. rufipes* gut of our study, explaining the higher concentration of this genus in CNV field.

On the whole, it is certain that the number of taxa (families and genera) detected in the gut microbiota of *P. rufipes*, in both farming conditions, is generally lower than that reported in the study involving *P. melas italicus* and in the large number of studies involving the gut microbiota of social insects like bees^7,50,51^. We can speculate that, in our study, involving solitary insects collected in fields, strain acquisition from other insects or other sources, like flowers in bees, may be hampered, thus reducing the whole number of taxa.

## Methods

### Area of study

The study was conducted at the Council for Agricultural Research and Economics – Research Centre for Vegetable and Ornamental Crops (CREA-OF) in Monsampolo del Tronto, Marche Region, (latitude 42° 53′ N, longitude 13° 48′ E). The climate of the locality is classified as thermo-Mediterranean, characterized by mild–cool winters and hot summers with cumulative annual precipitation and mean annual temperature (in 2018) of 789 mm and 15.7 °C, respectively. The soil is classified as Typic Calcixerepts fine-loamy, mixed thermic^52^.

Carabidae collections for gut microbiome analysis were carried out in two experimental areas, one within an organic rotation (42° 53.04’ N, 13°47.88’ E) managed since 2001 (organic) according to European legislation for organic farming, while the other within a neighboring (42° 53.11’ N, 13° 47.78’ E) conventional rotation (conventional). Carabids were sampled in tomato fields (*Solanum lycopersicum* L., variety SAAB-CRA) belonging to the different management regimes (organic [ORG] vs conventional [CNV]). The same crop was selected to avoid potential crop effects and to standardize comparisons between the microbiota of the different managements; tomato crop was selected for its local and regional economic importance.

Descriptions of the agronomic practices for each management are provided in Table 1. The Organic management is part of the MOnsampolo VEgetable (MOVE) organic long-term field experiment and is characterized by a four-year crop rotation^32^. In particular, the in-line roller-crimper technique^33^ was used for flattening strips of faba bean (*Vicia faba* L.) after fresh pod harvest. In this way, a conservation tilling strategy and strip cropping cultivation of tomato for the fresh market and faba bean for dry grain harvest was realized. This site was selected because cultivation of organic and conventional in the same experimental farm should prevent potential differences of microbiota due by local factors. Also, MOVE long-term has provided in the last years standardized comparison between organic and conventional^27,32,33^, including faunistic analysis of soil arthropods^34^.

### Sampling

Carabidae collection was employed using the standard pit-fall method. Each single collecting apparatus consisted of a double plastic cup system, between which a cavity filled with commercial vinegar was left, with the purpose to increase attractiveness of traps^53^. The bottom of the upper glass was perforated in order to ensure the diffusion of the vinegar smell. Traps were protected by saucers to prevent the filling of water in case of rain and to hide the trap from vertebrates that could be attracted by the captured arthropods or by the capture liquid.

Five collecting stations were set in each management, each station consisting of 2 double trap system (for a total of 10 double traps for each sampling and management) connected by a plastic barrier to increase the Carabid collection^35^. In each management, one collecting station was settle in the field border, while the other four stations within tomato crop.

Carabids were sampled from early June to the end of August in each management. Traps were activated for one day, to collect alive Carabid specimens. Each trap was checked early in the morning (between 8:00 and 9:00) by collecting Carabids fallen inside the cups. Insect were then placed in single plastic Falcons and immediately transported to the laboratory where were frozen (− 80 °C) until further analysis.

### Carabid dissection for gut extraction

The samples were surface sterilized by immersion in ethanol (70%) for 1 min and washed in solution of Sterile Ringer’s solution (CaCl_2_ 3 mM, KCl 182 mM, NaCl 46 mM, Tris Base 10 mM; pH 7,2). After, the legs were removed, and the specimens were pinned in the left corner between the pronotum and the terminal part of the abdomen; then elytra and metathoracic wings were also taken off. The specimens were re-washed in Sterile Ringer’s solution; the exoskeleton of pronotum, mesonotum and metanotum was cut lengthwise and the abdominal cuticle opened laterally, with the intention to leave intact the gut canal without damaging it. The entire gut (including foregut, midgut and hindgut) was extracted and clamped with microvascular clamps (1.0–2.5 mm, S&T’ AG, Neuhausen am Rheinfall, Switzerland) to prevent the escape of the intestinal contents. Finally, the entire gut was then externally disinfected in ethanol (70%), washed in Phosfate Buffer Saline (PBS) pH 7.2, placed in sterile 1.5 ml tubes and stored at − 80 °C.

### DNA extraction

DNA was extracted from the gut content by mechanical and subsequent chemical lysis, using the Quick-DNA Tissue / Insect Microprep Kit (Zymo Research, Irvine, CA, USA) following the instructions. Then, the DNA extracted was quantified by spectrophotometric method (Infinite^®^ 200; TECAN, Männedorf, Switzerland). The extracted DNA was stored at − 20 °C until use, and then it was diluted to 5 ng/μl in a final volume of 20 μl (see annex).

### qPCR analysis

qPCR on total bacteria (eubacteria) was carried out using the StepOne™ Real-Time PCR System (Applied Biosystem, Foster City, CA, USA). The amplification reactions were performed in triplicate in a total volume of 20 μl with SYBR Green chemistry. Each reaction consisted of 10 μl of Fast SYBR Green Master Mix (Applied Biosystem), 0.1 ÷ 0.3 μl of each primer, 2 μl of DNA and water up to the final volume of 20 μl. Amplification reactions were conducted on the basis of the following protocol: Activation 95 °C for 30 s, 40 cycles of denaturation at 95 °C and annealing at 59.0 °C, final melting curve with a temperature gradient increase of 0.3 °C from 60 to 95 °C. Data are expressed as “Log 16S rRNA copies/intestine” for the respective targets. qPCR results are reported in the Supplementary Materials file together with Figure S1.

### Amplicon based NGS Sequencing

DNA was extracted from 29 *P. rufipes* samples. All samples were subjected to NGS analysis on Illumina MiSeq platform, but eight failed. The V3-V4 region of 16S rRNA was amplified and sequenced. The amplicons, approximately 460 bp, were generated using the primers described in Takahashi et al.^54^ (Table 2). Bioassays were done using a 50 μl PCR amplification mix consisting of 25 μl HiFi KAPA HiFi HotStart ReadyMix (KAPA Biosystem, Woburn, MA, USA), a concentration of primer of 0.2 μM, H2O molecular grade and 5 μl of DNA (50 ng/μl). The amplification was performed using the following steps: activation at 95 °C for 3 min followed by 27 cycles at 95 °C for 30 s, 55 °C for 30 s and 72 °C for 30 s, followed by a step of final elongation at 72 °C for 5 min. The PCR products were purified using the AMPure beads XP purification system (Backman Coulter, UK) following the instructions for Illumina 16S Ribosomial RNA Gene Amplicon. Amplicons were then barcoded using the Nextera XT v2 Index Kit D (Illumina, SanDiego, CA, USA). The following program was used for the second PCR amplification: 95 °C for 3 min followed by 8 cycles at 95 °C for 30 s, 55 °C for 30 s and 72 °C for 30 s and one final elongation at 72 °C for 5 min. Then, a purification step using the AMPure beads XP purification system protocol (Beckman Coulter, UK) was employed. The libraries were quantified by means of the Qubit 2.0 Fluorometer (Invitrogen, Life Technologies, Carlsbad, CA, USA); then, after pooling them in equimolar quantities (4 nM), libraries were sequenced on the MiSeq platform (2 × 300 pair-end sequencing). The sequencing process was conducted by Bio-Fab research s.r.l. (Rome, Italy) using V3 chemistry. Due to the low number of reads, samples GQ, 25Q, 28Q, 40Q and 43Q were omitted from the analysis. For the statistical analysis, samples were rarefied at 33,717 joint reads. The taxonomical assignment of the remaining 16 samples produced 21,559 OTUs at 97% similarity based on SILVA v132 database. The Obtained OTUs were grouped in 798 taxonomical assignations at species level. NGS normalized relative abundance, at phylum, family and genus level are reported in Supplementary Materials (Tables S2–S4).

**Table 2:** List of PCR primer used in this work.

	Primer Name	Sequence (5′-3′)	Amplicon size	Reference
Eubacteria	Eub338-F	ACTCCTACGGGAGGCAGCAG	200	Muyzer et al. (1993)
	Eub518-R	ATTACCGCGGCTGCTGG		
Illumina adapter—V3-V4 Region of 16S rRNA gene	Pro341-F	*AATGATACGGCGACCACCGAGATCTACACTCTTTCCCTACACGACGCTCTTCCGATCTCCTACGGGAGGCAGCAG*-**CCTACGGGNGCASCAG**	560	Takahashi et al. (2014)
	Pro805-R	*CAAGCAGAAGACGGCATACGAGATNNNNNNGTGACTGGAGTTCAGACGTGTGCTCTTCCGATCT*-**GACTACNVGGGTATCTAATC**C		

### Bioinformatic analysis

The sequences of approximately 300 bp obtained in forward and reverse were assembled using FLASH^55^. The sequences were analyzed using QIIME ver. 1.9.1^56,57^, including a filter based on a quality score > 25 and removing barcodes with mismatch and sequences less than the threshold length. Denoising, detection of chimeras and grouping into operational taxonomic units (OTUs) (97% equality) were performed using USEARCH version 7^58^. The OTU sequences were aligned using the PyNAST^59^ and the classification was conducted using the SILVA SSU Ref database release 128^60,61^. The analysis of the indices of biodiversity was carried out using QIIME, especially the script “Core_diversity_analysis.py”; the phylogenetic classification of OTUs was conducted with the script “make_phylogen.py” (fasttree). α-diversity was assessed using the following metrics: Chao, Observed OTU and PD whole tree; β-diversity was evaluated using the weighted and unweighted UniFrac metric^62^. Finally, rarefied biom tables obtained from NGS bioinformatic analysis were further adjusted according to Raymann et al.^63^: the absolute abundance of each bacteria species was obtained by multiplying relative abundance data of each sample to the corresponding qPCR total bacterial amount. The values obtained for each genus were normalized by the 16S rRNA gene copy number typical of each microbial genus, according to database^1,2^ or to the genomes retrieved from the NCBI ReSeq database.

### Statistical analysis

Statistical analysis for qPCR and NGS data (α-diversity and taxon analysis) was performed with the R software according to Baffoni et al.^10^. β-diversity index was based on the QIIME statistical elaboration reports. Graphs were generated with ggplot2 and ggpubr. A comparison of the concentration of Eubacteria concentration between each farm management (ORG against CNV) was employed using the non-parametric median test.

A permutational analysis of variance (PERMANOVA) was used to preliminary compare absolute concentrations of microbiome taxa (phyla, families, genera) in organic against conventional in order to detect potential differences in microbiome taxa composition between farm management practices. Multivariate correspondence analysis (CA) was performed to ordinate organic and conventional samples according to absolute concentration of microbiome taxa. Families and genera were selected for multivariate analysis when relative abundance of at least one sample was > 4%. The aim of this analysis was to describe sample variability and to correlate microbiome taxa with each farm management (ORG or CNV). Hereafter, taxa (families and genera) showing the greatest correlation with each farm management were further analyzed with non-parametric median test (*P* < 0.05) in order to compare absolute concentrations of each taxon between ORG and CNV; median test was also performed to compare genera belonging to the families that previously showed differences between ORG and CNV. Finally, Indicator Value Analysis (indVal) was performed with R package “labdsv”^64^, to in order to assess taxa specificity to experimental conditions.

### Ethical approval

Ethical review and approval were waived for this study, because the Italian law does not require an ethical approval for tests performed on arthropods with exceptions of cephalopods according to the Italian D.L. 4 March 2014 n. 26, and Italian implementing decree following the European regulation 2010/63/UE.

## Supplementary Information

Supplementary Information.

## Data Availability

NGS 16S rRNA sequence data have been deposited at [NCBI repository Sequence Read Archive (SRA) and are publicly available as of the date of publication. Accession numbers are listed in the key resources table. Bio Project No. PRJNA798890, accession numbers SAMN25144112–SAMN25144116; SAMN25144118–SAMN25144135. Supplementary data, including excel files of elaborated data obtained from qPCR for target microbial groups and NGS data categorized at different taxonomical level can be found at this repository: https://data.mendeley.com/datasets/hdwyxx8h6k/1.
