# Supplementary material for: The ground beetle Pseudoophonus rufipes gut microbiome is influenced by the farm management system
Source: Sci Rep. 2022 Dec 31;12:22638. doi: 10.1038/s41598-022-25408-7 (PMC9805440; doi:10.1038/s41598-022-25408-7)
Supplement: Supplementary file 1 — Supplementary Information. [file 41598_2022_25408_MOESM1_ESM.pdf]

Supplementary material of:

The ground beetle *Pseudoophonus rufipes* gut microbiome is influenced by the farm management system

Authors: **Serena Magagnoli[1†], Daniele Alberoni[1†*], Loredana Baffoni[1], Antonio Martini[1], Francesca Marini[1], Diana Di Gioia[1], Martina Mazzon[1], Claudio Marzadori[1]., Gabriele Campanelli[2], Giovanni Burgio[1].**

Affiliations:

[1] Dipartimento di Scienze e Tecnologie Agro-Alimentari (DISTAL), Università di Bologna, Viale Fanin 42, 40127, Bologna, Italy;

[2] Consiglio per la ricerca in agricoltura e l'analisi dell'economia – Orticoltura e Florovivaismo (CREA-OF) - Sede di Monsampolo del Tronto, via Salaria 1, Italy

Correspondence:

[*] Daniele Alberoni: daniele.alberoni@unibo.it

qPCR results

Concentrations of Eubacteria did not show any difference between ORG and CNV (P>0.05) and were Log 6.07 ±0.20 and 6.10 ±0.32 of 16S rRNA copies/intestine, respectively.

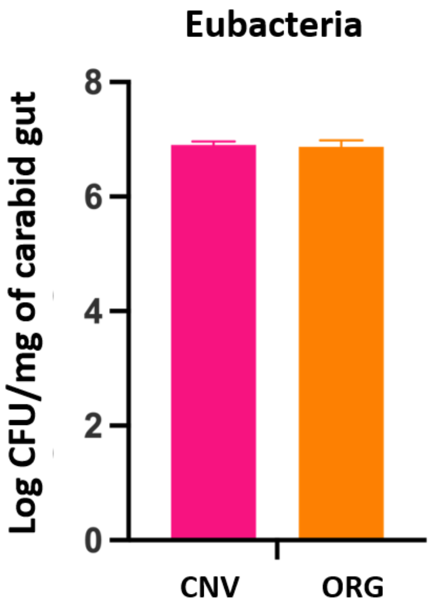

Fig. S1. qPCR: Quantification of total bacteria (Eubacteria), Data are expressed in Log of CFU/ mg of carabid gut.

Supplementary tables

Table S1: β-diversity output

**Group 1**	**Group 2**	***p***
weighted UniFrac		
ORG *vs* ORG	CNV *vs* CNV	0.45
unweighted UniFrac		
ORG *vs* ORG	CNV *vs* CNV	0.0017

Table S2: raw data at Phyla level

	CNV						ORG									
	C	D	E	F	H	I	15Q	23Q	24Q	26Q	27Q	29Q	30Q	36Q	39Q	45Q
**Actinobacteria**	2,798	3,066	0,036	0,044	0,811	0,007	0,042	0,011	0,075	0,83	4,354	3,158	0,129	5,915	58,05	0,218
**Bacteroidetes**	0,079	3,572	0,005	0,049	0,008	0,004	0,006	0,005	0,032	0,029	0,301	0,279	0,015	0,004	0,041	0,01
**Firmicutes**	25,75	30,82	0,215	48,22	0,389	0,169	0,043	0,205	0,121	0,602	2,974	1,509	0,134	1,66	12,93	0,085
**Proteobacteria**	61,81	23,89	0,528	28,62	60,47	52,51	0,013	92,25	0,041	0,187	0,227	0,282	0,023	1,627	5,606	0,043
**Tenericutes**	0,251	10,65	98,59	0,078	34,51	45,95	99,65	6,793	99,12	97,23	89,49	92,62	99,37	82,11	0,133	99,12
**other**	9,319	27,99	0,622	23	3,809	1,357	0,25	0,735	0,606	1,121	2,653	2,149	0,326	8,683	23,24	0,523

Table S3: raw data at Family level

	CNV						ORG									
	C	D	E	F	H	I	15Q	23Q	24Q	26Q	27Q	29Q	30Q	36Q	39Q	45Q
Bifidobacteriaceae	0,000	1,755	0,006	0,013	0,011	0,002	0,003	0,010	0,014	0,562	0,367	1,620	0,004	5,886	57,990	0,057
Propionibacteriaceae	2,798	1,312	0,030	0,031	0,800	0,005	0,039	0,002	0,062	0,268	3,987	1,538	0,124	0,029	0,058	0,161
Bacteroidaceae	0,016	1,909	0,001	0,009	0,003	0,001	0,000	0,001	0,014	0,001	0,072	0,102	0,007	0,002	0,022	0,003
Rikenellaceae	0,063	1,663	0,005	0,039	0,005	0,003	0,006	0,004	0,018	0,028	0,229	0,177	0,007	0,002	0,019	0,008
Staphylococcaceae	0,065	0,108	0,006	0,037	0,020	0,001	0,001	0,000	0,008	0,134	0,198	0,235	0,002	0,460	7,685	0,019
Enterococcaceae	23,686	0,245	0,011	26,127	0,095	0,060	0,000	0,005	0,007	0,089	0,002	0,025	0,014	0,047	1,529	0,001
Enterobacteriaceae	14,072	22,867	0,096	13,246	0,429	2,308	0,007	4,409	0,039	0,085	0,021	0,061	0,017	1,263	1,228	0,017
Eubacteriales	0,000	2,003	0,000	0,016	0,018	0,000	0,001	0,000	0,012	0,031	0,171	0,077	0,016	0,000	0,004	0,000
Lactobacillaceae	0,237	0,850	0,183	20,835	0,024	0,007	0,019	0,036	0,017	0,153	0,106	0,221	0,008	1,106	3,575	0,030
Streptococcaceae	1,011	0,102	0,001	1,097	0,005	0,086	0,000	0,148	0,020	0,067	0,115	0,087	0,004	0,013	0,016	0,008
Oscillospiraceae	0,637	24,743	0,011	0,074	0,182	0,012	0,015	0,013	0,047	0,126	2,011	0,754	0,085	0,031	0,112	0,025
Wolbachieae	0,000	0,022	0,003	0,000	52,700	50,200	0,000	0,000	0,000	0,000	0,052	0,047	0,000	0,000	0,000	0,006
Hepatincolaceae	0,084	0,000	0,000	0,000	0,000	0,000	0,003	87,836	0,000	0,034	0,003	0,010	0,000	0,000	0,000	0,000
Chromobacteriaceae	0,086	0,000	0,000	0,000	0,000	0,000	0,000	0,000	0,000	0,000	0,000	0,000	0,000	0,000	0,000	0,000
Neisseriaceae	47,477	0,024	0,002	0,008	0,014	0,001	0,001	0,006	0,002	0,001	0,123	0,017	0,004	0,002	0,018	0,004
Morganellaceae	0,052	0,012	0,000	13,349	0,001	0,000	0,000	0,000	0,000	0,000	0,000	0,000	0,000	0,000	0,000	0,000
Yersiniaceae	0,034	0,005	0,426	1,715	7,323	0,000	0,001	0,000	0,000	0,001	0,004	0,012	0,003	0,001	0,001	0,000
Spiroplasmataceae	0,251	8,206	98,594	0,078	34,513	45,955	99,643	6,793	99,124	97,230	89,384	92,612	99,367	82,111	0,133	99,121
Lachnospiraceae	0,064	1,896	0,001	0,028	0,017	0,000	0,001	0,001	0,004	0,001	0,125	0,044	0,003	0,002	0,002	0,001
Ruminococcaceae	0,049	0,875	0,002	0,004	0,028	0,003	0,004	0,002	0,007	0,001	0,246	0,067	0,001	0,001	0,008	0,002
Acetobacteraceae	0,000	0,000	0,000	0,298	0,000	0,000	0,000	0,000	0,000	0,051	0,004	0,133	0,000	0,362	2,711	0,016
Campylobacteraceae	0,000	0,965	0,000	0,000	0,002	0,000	0,000	0,000	0,000	0,015	0,020	0,002	0,000	0,000	0,000	0,000
Gammaproteobacteria	0,000	0,000	0,000	0,000	0,000	0,000	0,000	0,000	0,000	0,000	0,000	0,000	0,000	0,000	1,649	0,000
Mollicutes unclassified	0,000	2,446	0,000	0,000	0,002	0,000	0,003	0,000	0,000	0,000	0,107	0,012	0,006	0,000	0,000	0,000
Other	9,319	27,995	0,622	22,995	3,809	1,357	0,250	0,735	0,606	1,121	2,653	2,149	0,326	8,683	23,242	0,523

Table S4: raw data at Genus level

	CNV						ORG									
	C	D	E	F	H	I	15Q	23Q	24Q	26Q	27Q	29Q	30Q	36Q	39Q	45Q
Acetobacteraceae unclassified	0,000	0,000	0,000	0,298	0,000	0,000	0,000	0,000	0,000	0,051	0,004	0,133	0,000	0,362	2,711	0,016
Alistipes	0,063	1,663	0,005	0,039	0,005	0,003	0,006	0,004	0,018	0,028	0,229	0,177	0,007	0,002	0,019	0,008
Anaerotruncus	0,000	4,934	0,000	0,016	0,000	0,000	0,000	0,000	0,003	0,009	0,252	0,093	0,006	0,000	0,008	0,003
Apilactibacillus	0,000	0,000	0,000	0,000	0,000	0,000	0,000	0,000	0,000	0,000	0,001	0,002	0,000	0,008	0,076	0,000
Aquaspirillum	0,086	0,000	0,000	0,000	0,000	0,000	0,000	0,000	0,000	0,000	0,000	0,000	0,000	0,000	0,000	0,000
Bacteroides	0,016	1,909	0,001	0,009	0,003	0,001	0,000	0,001	0,014	0,001	0,072	0,102	0,007	0,002	0,022	0,003
Bifidobacteriaceae unclassified	0,000	0,000	0,000	0,005	0,000	0,000	0,000	0,000	0,000	0,011	0,000	0,039	0,000	0,132	1,224	0,000
Bifidobacterium	0,000	1,755	0,006	0,008	0,011	0,002	0,003	0,010	0,014	0,524	0,364	1,491	0,004	5,423	53,625	0,056
Campylobacter	0,000	0,965	0,000	0,000	0,002	0,000	0,000	0,000	0,000	0,015	0,020	0,002	0,000	0,000	0,000	0,000
Citrobacter	0,089	0,235	0,030	3,046	0,055	0,624	0,000	0,003	0,002	0,021	0,003	0,018	0,001	0,078	0,487	0,004
Enterobacter	0,021	1,706	0,023	0,947	0,029	0,014	0,000	0,214	0,000	0,005	0,001	0,005	0,002	0,014	0,013	0,001
Enterobacteriaceae unclassified	11,144	20,550	0,042	9,188	0,344	1,668	0,007	4,122	0,035	0,056	0,016	0,037	0,013	0,910	0,718	0,012
Enterococcus	3,906	0,121	0,004	9,357	0,043	0,002	0,000	0,002	0,003	0,026	0,001	0,010	0,003	0,017	0,102	0,001
Faecalibacterium	0,032	3,909	0,001	0,002	0,037	0,000	0,002	0,002	0,017	0,018	0,231	0,126	0,012	0,001	0,007	0,003
Flavonifractor	0,007	0,972	0,002	0,000	0,005	0,000	0,001	0,000	0,000	0,002	0,131	0,062	0,003	0,000	0,000	0,003
Gardnerella	0,000	0,000	0,000	0,000	0,000	0,000	0,000	0,000	0,000	0,027	0,003	0,090	0,000	0,330	3,142	0,002
Hepatincola	0,084	0,000	0,000	0,000	0,000	0,000	0,003	87,836	0,000	0,034	0,003	0,010	0,000	0,000	0,000	0,000
Intestinimonas	0,000	2,003	0,000	0,016	0,018	0,000	0,001	0,000	0,012	0,031	0,171	0,077	0,016	0,000	0,004	0,000
Klebsiella	0,000	0,000	0,000	0,000	0,000	0,000	0,000	0,000	0,000	0,001	0,000	0,000	0,000	0,001	0,003	0,000
Koukoulia	0,000	0,000	0,000	0,000	0,000	0,000	0,000	0,000	0,000	0,000	0,000	0,000	0,000	0,000	1,649	0,000
Lachnospiraceae unclassified	0,064	1,896	0,001	0,028	0,017	0,000	0,001	0,001	0,004	0,001	0,125	0,044	0,003	0,002	0,002	0,001
Lacticaseibacillus	0,000	0,000	0,000	0,000	0,000	0,000	0,000	0,000	0,000	0,000	0,021	0,005	0,000	0,000	0,003	0,000
Lactiplantibacillus	0,003	0,003	0,002	0,122	0,003	0,000	0,001	0,000	0,001	0,056	0,004	0,059	0,000	0,172	0,121	0,002
Lactobacillaceae (unclassified lactobacillus)	0,125	0,275	0,007	1,200	0,014	0,007	0,017	0,011	0,016	0,034	0,031	0,032	0,008	0,141	1,927	0,014
Lactobacillus	0,000	0,234	0,000	0,000	0,000	0,000	0,000	0,000	0,000	0,004	0,039	0,044	0,000	0,000	0,001	0,001
Lactococcus	0,969	0,020	0,000	1,097	0,000	0,085	0,000	0,148	0,009	0,059	0,003	0,008	0,001	0,012	0,016	0,003
Latilactobacillus	0,012	0,000	0,001	0,002	0,001	0,000	0,000	0,000	0,000	0,057	0,004	0,075	0,000	0,308	0,285	0,012
Leclercia	0,002	0,000	0,000	0,012	0,000	0,000	0,000	0,000	0,000	0,000	0,000	0,000	0,000	0,000	0,001	0,000
Levilactobacillus	0,070	0,000	0,000	17,215	0,005	0,000	0,000	0,000	0,000	0,000	0,001	0,000	0,000	0,000	0,000	0,000
Ligilactobacillus	0,000	0,315	0,000	0,000	0,000	0,000	0,000	0,000	0,000	0,000	0,003	0,004	0,000	0,000	0,000	0,000
Mollicutes unclassified	0,000	2,446	0,000	0,000	0,002	0,000	0,003	0,000	0,000	0,000	0,107	0,012	0,006	0,000	0,000	0,000
Neisseriaceae unclassified	46,844	0,019	0,001	0,000	0,012	0,001	0,001	0,003	0,000	0,001	0,121	0,016	0,002	0,002	0,002	0,003
Oscillibacter	0,190	1,073	0,002	0,005	0,000	0,000	0,000	0,001	0,001	0,015	0,141	0,028	0,000	0,000	0,000	0,002
Other	9,319	27,995	0,622	22,995	3,809	1,357	0,250	0,735	0,606	1,121	2,653	2,149	0,326	8,683	23,242	0,523
Propionibacterium	2,798	1,312	0,030	0,031	0,800	0,005	0,039	0,002	0,062	0,268	3,987	1,538	0,124	0,029	0,058	0,161
Proteus	0,052	0,012	0,000	13,349	0,001	0,000	0,000	0,000	0,000	0,000	0,000	0,000	0,000	0,000	0,000	0,000
Raoultella	2,817	0,377	0,000	0,053	0,001	0,001	0,000	0,069	0,002	0,001	0,000	0,000	0,000	0,260	0,006	0,000
Ruminococcaceae unclassified	0,049	0,875	0,002	0,004	0,028	0,003	0,004	0,002	0,007	0,001	0,246	0,067	0,001	0,001	0,008	0,002
Ruminococcus	0,226	8,748	0,007	0,020	0,100	0,005	0,009	0,007	0,017	0,073	0,753	0,359	0,058	0,011	0,019	0,008
Serratia	0,034	0,005	0,426	1,715	7,323	0,000	0,001	0,000	0,000	0,001	0,004	0,012	0,003	0,001	0,001	0,000
Snodgrassella	0,007	0,005	0,002	0,008	0,001	0,000	0,001	0,003	0,002	0,000	0,003	0,001	0,001	0,000	0,016	0,001
Spiroplasma	0,251	8,206	98,594	0,078	34,513	45,955	99,643	6,793	99,124	97,230	89,384	92,612	99,367	82,111	0,133	99,121
Staphylococcus	0,065	0,108	0,006	0,037	0,020	0,001	0,001	0,000	0,008	0,134	0,198	0,235	0,002	0,460	7,685	0,019
Streptococcus	0,042	0,082	0,001	0,000	0,005	0,001	0,000	0,000	0,011	0,008	0,112	0,079	0,002	0,002	0,000	0,005
Subdoligranulum	0,181	5,107	0,000	0,031	0,041	0,007	0,003	0,004	0,009	0,009	0,503	0,087	0,006	0,020	0,078	0,006
Vagococcus	19,780	0,124	0,007	16,770	0,052	0,059	0,000	0,003	0,004	0,063	0,001	0,016	0,011	0,029	1,427	0,001
Vitreoscilla	0,626	0,000	0,000	0,000	0,000	0,000	0,000	0,000	0,000	0,000	0,000	0,000	0,000	0,000	0,000	0,000
Weissella	0,027	0,023	0,173	2,296	0,002	0,000	0,000	0,025	0,000	0,003	0,000	0,000	0,000	0,478	1,162	0,000
Wolbachia	0,000	0,022	0,003	0,000	52,700	50,200	0,000	0,000	0,000	0,000	0,052	0,047	0,000	0,000	0,000	0,006

The same data in excel files format can be found at this repository: https://data.mendeley.com/datasets/hdwyxx8h6k/1.
